# The Content of COVID-19 Information Searches and Vaccination Intention: An Implication for Risk Communication

**DOI:** 10.1017/dmp.2022.257

**Published:** 2022-11-03

**Authors:** Ayokunle A. Olagoke, Brenikki Floyd, Comfort T. Adebayo, Ayomide Owoyemi, Ashley M. Hughes

**Affiliations:** 1 School of Health and Kinesiology, University of Nebraska at Omaha, NE, USA; 2 Division of Community Health Sciences, School of Public Health, University of Illinois at Chicago, IL, USA; 3 Department of Communication Studies, Towson University, Maryland, USA; 4 Department of Biomedical and Health Information Science, University of Illinois at Chicago, IL, USA; 5 Center of Innovation for Complex Chronic Healthcare, Edward Hines JR VA Hospital, Hines, IL, USA

**Keywords:** COVID-19, information search, risk communication, vaccination intention

## Abstract

**Objective:**

The main objective of this study was to examine the association between COVID-19 information search activities and vaccination intention.

**Methods:**

Cross-sectional data were collected using online surveys. Independent variables included COVID-19 information search on the (1) science of viral effects of COVID-19 on the body, (2) origin of COVID-19, (3) symptoms and outcomes, (4) transmission and prevention, (5) future outbreak, and (6) policies/procedures to follow. The outcome variable was vaccination intention. A multivariable regression analysis was conducted.

**Results:**

Participants (N = 501) had a mean age of 32.44 ± 11.94 years, were 55.3% female, and 67.9% were white. Most COVID-19 information searches were on symptoms and outcomes (77.7%) and policies/procedures to follow (69.9%). Intention to vaccinate against COVID-19 was higher among participants who searched for information on the science of viral effects of COVID-19 on the body (*β* = 0.23, 95% CI: 0.03-0.43; *P* = 0.03) and policies/procedures to follow (*β* = 0.24, 95% CI: 0.03-0.41; *P* = 0.02).

**Conclusions:**

People who searched for information about (1) the science of viral effects of COVID-19 and (2) policies/procedures recommendations also reported higher vaccination intention. Risk communication seeking to increase vaccination should meet the consumers’ information demand by prioritizing the scientific rationale for COVID-19 vaccination and by clarifying what policies/procedures are recommended.

The coronavirus disease (COVID-19) is caused by severe acute respiratory syndrome coronavirus 2 (SARS-CoV-2). COVID-19 is an infectious disease of national and global importance. It was rated the second leading cause of death in 2020 among people under age 85 years.^1^ This infection’s transmissibility and high fatality have prompted government bodies, organizations, and the public to rapidly send and receive COVID-19-related health information, which is a significant step in crisis response and mitigation.^2,3^ People have searched for information from multiple sources such as government websites, medical websites, family members, scientists or researchers, social media, and television.^4,5^ These sources have disseminated information such as the etiology and epidemiology of the virus, origin of the disease, symptoms, and outcomes of the infection, its transmission, and prevention (eg, vaccination), the risk of a future outbreak, policies, and procedures to follow (such as mask mandates). An analysis of a Google search for 2021 shows that COVID-19-related keywords dominated the search engine in 2021,^6,7^ indicating a massive demand for COVID-19-related health information.

However, the demand for up-to-date information, the urgency, and uncertainty around COVID-19 has left little or no time to empirically test how these information search behaviors are associated with COVID-19-related outcomes such as vaccination. The complementarity theory^8^ suggests that people search for specific information based upon the functions relevant to them. Therefore, it is essential to know what information people are searching for regarding COVID-19 to understand their (1) information needs and (2) information-seeking behavior, thereby providing rapid evidence to inform policymaking and risk communication. This knowledge can guide the development of tailored communication resources based on the consumers’ information demand and can serve as an early-warning system linking information search activities to behavioral outcomes like vaccination.^9^

Despite the availability of an approved COVID-19 vaccine, acceptance has been suboptimal, with less than 50% of the eligible population in the United States fully vaccinated with booster shots.^10,11^ A major reason for low uptake has been cited as the misunderstanding of the information surrounding vaccination^12^ and the spread of misinformation.^13–15^ Therefore, the knowledge of information search patterns can also offer clarity to areas where information consumers experience messaging needs and misunderstanding, which is a strong predictor of vaccination intention.^12^

There have been gaps in COVID-19 information-seeking research. First, most studies have emphasized information dissemination (not search) and vaccination outcomes of the search. Information dissemination and search are not the same. An example is a randomized controlled trial that presented participants with specific health messages to test how their exposure to the messages influenced their vaccination decisions.^16,17^ The downside of this approach is that it is a controlled/manipulated experiment and does not observe participants’ selective exposure to information. Hence these types of study can test information dissemination and not information search since the latter is initiated by the consumers and not the information providers. Second, past studies have reported COVID-19 information search in isolation without connecting it to behavioral outcomes.^18,19^ They have explored COVID-19-related search interest, sources, search frequency, information processing, and comprehension. Very few studies have connected COVID-19 information search to vaccination outcomes like willingness to be vaccinated. In a recent study where COVID-19 search patterns were examined on Google, they linked these patterns to economic outcomes and suggested that future studies connect COVID-19 information search patterns (not dissemination) to health-related outcomes.^20^ Third, existing studies have also emphasized the science and health-related aspect of COVID-19 information search with less inquiry into the communication strategies by which information content is made available to users.^21^

This current study explores the association between COVID-19 information search activities and vaccination intention. Previous studies have established that access to accurate and timely information is essential to increase vaccination.^22,23^ However, this study answers the question of *what type of COVID-19 information search activity is associated with COVID-19 vaccination*? The type and quality of information persons are exposed to can influence their risk perception and disease prevention strategy, hence their vaccination decision.^4^ Considering that COVID-19 vaccine misinformation has been identified as a significant threat to vaccination and there is a clarion call to spread factual COVID-19 information, this study offers a window into what type of information may be associated with vaccination intention.

## Methods

### Study Sample

This study enrolled participants via Prolific, an online crowdsourcing platform for researchers. Prolific has a diverse participant pool and generates high data quality. For example, participants from Prolific scored higher on attention checks, engaged in lesser deceitful behavior, and were able to reproduce existing results^24^ compared to other crowdsourcing platforms. The two eligibility criteria for enrolling in this study were (1) residing in the United States and (2) being age 18 years or older. Cross-sectional data were collected from 502 participants on March 22, 2020, using the Qualtrics online survey. Each participant received an incentive of $0.55 after survey completion. The Institution Review Board of the University of Illinois, Chicago, approved the study.

### Dependent Variable

COVID-19 vaccination intention was assessed using a single item that asked participants, “If there is a preventive vaccine against COVID-19, how likely are you to receive the vaccine?”^4^ Responses were reported on a 5-point Likert scale ranging from extremely unlikely (“1”) to extremely likely (“5”).

### Independent Variables

1. Search for the science of viral effect on the body: Participants were asked if they searched for information regarding the viral effect of COVID-19 on the body in the past 3 days. Response options were “yes” or “no.”
2. Search for the origin of the disease: Participants were asked if they searched for information regarding the origin of COVID-19 disease in the past 3 days. Response options were “yes” or “no.”
3. Search for symptoms and outcomes of having COVID-19: Participants were asked if they searched for information regarding the symptoms and outcomes of COVID-19 infection in the past 3 days. Response options were “yes” or “no.”
4. Search for transmission and prevention: Participants were asked if they searched for information regarding the transmission and prevention of COVID-19 infection in the past 3 days. Response options were “yes” or “no.”
5. Search for a future outbreak: Participants were asked if they searched for information regarding a future outbreak of COVID-19 infection in the past 3 days. Response options were “yes” or “no.”
6. Search for policies/procedures to follow: Participants were asked if they searched for information regarding COVID-19 infection policies/procedures to follow in the past 3 days. Response options were “yes” or “no.”

### Covariates

Covariates included sociodemographic characteristics, for example, participant’s age (continuous variable), race, and marital status (married, divorced, separated, widowed, or single). Socioeconomic status (SES) characteristics were assessed, including household income (<$20 000, $20 000 to <$35 000, $35 000 to <$50 000, $50 000 to <$75 000, $75 000 or more); employment status, and highest education level completed (less than high school, high school graduate, some college, college graduate, or higher). The most recent sources of information were assessed (government website, medical website, scientists or researchers, social media, television, and others).

### Statistical Analysis

Participants’ characteristics were analyzed using descriptive statistics such as frequencies and percentages, means (for normally distributed continuous variables), and standard deviations (SD). Models were tested using multivariable linear regressions. Model 1 added all 6 independent variables without any covariates to test their crude independent associations with the dependent variable (vaccination intention). Model 2 added sociodemographic factors (ie, age, race, sex, marital status, most recent information source). In Model 3, we included socioeconomic status variables (ie, household income, employment status, and highest education level). Missing values were addressed with listwise deletion since they were less than 5%.^25^ Effect sizes and their confidence intervals were reported.^26^ A *P*-value < 0.05 was considered to be statistically significant. Statistical analyses were performed using SAS version 9.4.

## Results

**Figure 1.**
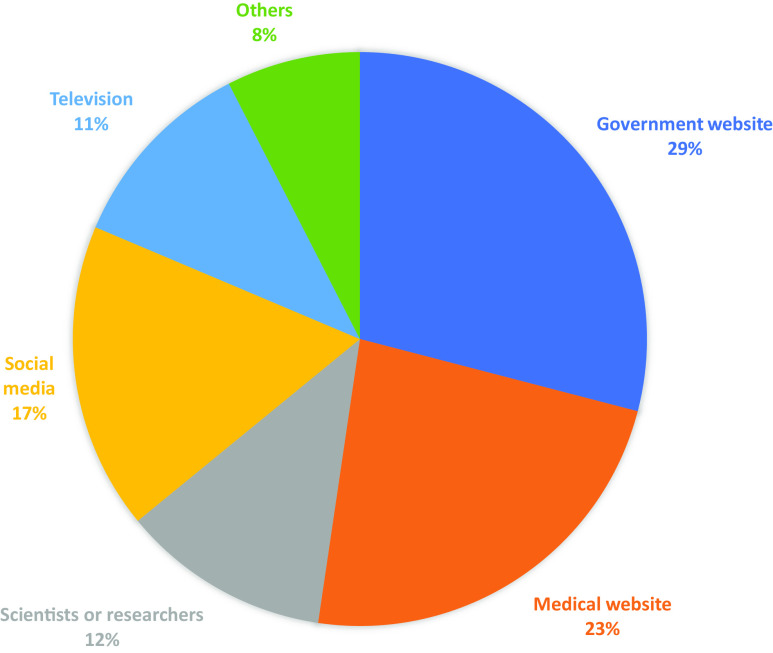
Most recent source of COVID-19 information.

After excluding 1 participant who failed the attention check (Table 1), the remaining 501 participants had a mean age of 32.44 ± 11.94 years. They were mostly females (55.3%), white (67.9%), unmarried (74.5%), with an education level of college graduates or higher (53.5%) and employed (56.5%). Selected responses to information search behaviors showed that 40% of the respondents searched for information on the science of viral effect of COVID-19 on the body, 23.5% for the origin of the disease, 77.7% for symptoms and outcomes, 69.3% for transmission and prevention, 47.6% for a future outbreak, and 69.9% for policies/procedures to follow. The most recent sources of information (Figure 1) were government websites (29%), medical websites (23%), social media (17%), scientists or researchers (12%), and television (11%). The mean vaccination intention was 4.2 ± 1.1, ranging from 1-5.

**Table 1.** Participants’ characteristics (N = 501)

Sample characteristics	Mean (SD)/N (%)
**Sociodemographic characteristics**	
Age	32.4 (11.9)
Sex	
Female	227 (55.3)
Male	224 (44.7)
Race	
White	340 (67.9)
Non-white	161 (32.1)
Marital status	
Not married	373 (74.5)
Married	128 (25.6)
**Socioeconomic status characteristics**	
Highest education	
Less than a college degree	233 (46.5)
College or more	268 (53.5)
Household income	
Less than $75 000	321 (64.3)
Over $75 000	178 (35.7)
Employment status	
Employed	275 (56.5)
Unemployed/retired/disabled/others	212 (43.5)
**Recent information search**	
The science of viral effect on the body	
Yes	199 (40)
No	266 (60)
Origin of the disease	
Yes	117 (23.5)
No	381 (76.5)
Symptoms and outcomes of having COVID-19	
Yes	387 (77.7)
No	111 (22.3)
Transmission and prevention	
Yes	345 (69.3)
No	153 (30.7)
Future outbreak	
Yes	237 (47.6)
No	261 (52.4)
Policies/procedures to follow	
Yes	348 (69.9)
No	150 (30.1)
**Outcome variables**	
Intention to vaccinate against COVID-19	4.2 (1.1)

Multivariable linear regression on vaccination intention (Table 2) in Model 1 showed that searching for policies/procedures was associated with high vaccination intention (β = 0.22, 95% CI: 0.01-0.42; *P* = 0.04). In Model 2, where we controlled for sociodemographic variables, searching for policies/procedures was reported among participants with high vaccination intention (0.25, 95% CI: 0.04-0.45; *P =* 0.02). In Model 3, where socioeconomic variables were added into the model, searching for the science of viral effect of COVID-19 on the body was positively associated with vaccination intention (0.23, 95% CI: 0.03-0.43; *P =* 0.03), and search for policies/procedures remained associated with high vaccination intention (0.24, 95% CI: 0.03-0.42; *P =* 0.02).

**Table 2.** Multivariable linear regression of information search on vaccine intention

Recent COVID-19 information search	Model 1		Model 2		Model 3	
	β (95% CI)	*P* value	β (95% CI)	*P* value	β (95% CI)	*P* value
Search for the science of viral effect on the body (ref = No)	0.17 (−0.03–0.37)	0.11	0.19 (−0.01–0.41)	0.06	**0.23 (0.03–0.43)**	**0.03**
Search for origin of the disease (ref = No)	0.11 (−0.12–0.33)	0.35	0.13 (−0.11–0.36)	0.27	0.11 (−0.13–0.33)	0.38
Search for symptoms and outcomes of having COVID-19 (ref = No)	−0.13 (−0.37–0.11)	0.26	−0.21 (−0.45–0.02)	0.08	−0.21 (−0.46–0.03)	0.08
Search for transmission and prevention (ref = No)	−0.14 (−0.35–0.07)	0.19	−0.16 (−0.37–0.06)	0.15	−0.13 (−0.35–0.09)	0.24
Search for future outbreak (ref = No)	0.09 (−0.09–0.28)	0.31	0.08 (−0.11–0.26)	0.43	0.05 (−0.14–0.23)	0.61
Search for policies/procedures to follow (ref = No)	**0.22 (0.01–0.42)**	**0.04**	**0.25 (0.04–0.45)**	**0.02**	**0.24 (0.03–0.41)**	**0.02**

## Discussion

This study builds on existing literature by going beyond the prevalence of COVID-19 information search to examine the content of the information search activities. We examined the association between COVID-19 information search activities and vaccination intention to understand the public’s information needs and how selective information exposure could relate to COVID-19 vaccination intention. Individuals who searched for information regarding the science of viral effect of COVID-19 also reported higher vaccination intention. A possible explanation for this can be drawn from the health belief model,^27^ which suggests that an understanding of the scientific mechanism of action of the virus could influence the socio-cognitive factors such as (1) a person’s risk perception (eg, perceived severity of COVID-19), (2) vaccine confidence (through an understanding of the science behind vaccine), and (3) level of worry. Heightened risk perception, vaccine confidence, and level of worry could lead to increased vaccination intention. For example, a study examining information-seeking behavior during COVID-19 found that risk-information seeking mediated the positive relationship between perceived severity and vaccination intention.^28^

We also found that information search activity on *policies/procedures to follow* was positively associated with vaccination intention. This was supported by Lampos’s (2021) work, who analyzed the Google search pattern during the COVID-19 outbreak and found searches for lockdown procedures/policies to be among the top searches.^29,30^ A possible explanation for these associations is that the public evaluates the magnitude of risk through government response and policies.^31^ Policies such as lockdown, restrictions on large gatherings (eg, restaurants, outpatient visits, and religious services) can elevate the COVID-19 risk awareness. Since health awareness is the first step toward disease prevention and health promotion,^32^ searching for policies/procedures could strengthen vaccination intention. Another explanation is that exposure to information about policies and procedures could build trust in the public. The policies/procedures may assure people that the government is taking the right steps to curb the pandemic. This confidence in the government’s approaches could influence their decision to follow other government’s recommendations such as vaccination. This is consistent with findings from a longitudinal study on vaccine hesitancy,^33^ where vaccine hesitancy decreased as public trust increased. Nevertheless, the association between policies and high COVID-19 vaccination may depend on the type of policies. Issues around the legality and ethics of some policies/mandates (eg, mask and vaccine mandate) could erode trust and heighten vaccine hesitancy.^34^

This study has clear implications for research and practice. We collected these data 1 month after the first COVID-19 case was detected in the United States. Therefore, our study findings show the immediate reaction to information search at the onset of the pandemic. Although COVID-19 has been here for almost 2 years now, the emergence of new variants and novel discoveries is causing more distressing situations that may recreate scenarios and information search patterns similar to the initial onset of the COVID-19 pandemic. We, therefore, offer insight into possible information demand and how this may be associated with vaccination. First, information about the science of viral effects should be disseminated to improve the public’s COVID-19 health literacy about the virus’s mechanism of action. Such information can be presented in simplified formats and plain languages to aid comprehension by the lay audience. An example is a COVID-19 health literacy intervention study that used physician-delivered messaging to increase COVID-19 information seeking intention, knowledge of viral effects, and risk reduction.^35^

Second, information such as the COVID-19 vaccine’s mechanism of action, the science of the viral effects, and clarifying policies/procedures may be relevant in increasing vaccination uptake. Although there is a safe and effective vaccine that can prevent COVID-19-related outcomes such as severe illness, hospitalization, and death, uptake has been suboptimal. Almost 40% of the eligible population is not fully vaccinated as of January 2022.^10^ COVID-19 vaccination disparity is even more pronounced among minority racial/ethnic groups such as African Americans and Hispanics.^36^ According to the Risk Information Seeking and Processing (RISP) model, affective responses, perceived information insufficiency, and perceived current knowledge predict online risk information search activities, leading to prevention intent.^37^ Hence, COVID-19 vaccination communication should be designed to meet people in the area of their expressed information needs (such as scientific rationale for the recommended COVID-19 policies/procedures to follow) as identified in this study to increase vaccination uptake.

Third, there is a need to ensure equilibrium in presenting COVID-19 information such that, as people consume information about the science and spread of the virus (which can be depressing), they are also provided with information about how they can be protected against the virus. In a recent study, cyberchondria (excessive worry over one’s health due to repeated Internet searches) mediated the relationship between fear of COVID-19 and vaccination intention.^30^ Public health communicators and media regulators should pay attention to the mental health of information consumers by counterbalancing threatening messages with empowering messages that will boost the consumers’ self-efficacy to take steps to protect them against COVID-19.

### Study Limitations

Despite the important contributions of our study, it is not without limitations. First, it is a cross-sectional study and could not establish temporal ordering. Vaccination intention may precede information seeking and not the other way round. However, several longitudinal studies have established that information seeking is necessary to predict intention in the multiple stages of behavioral change.^38^ Therefore, it is important to test our findings longitudinally in the context of COVID-19 infection and vaccination. Second, most of our measures were single items that could have introduced some elements of misclassification bias. However, our findings are consistent with previous studies that analyzed Google trends/searches^7,20^ and our use of single-item questions may lessen survey burden, which survey takers have reported during COVID-19.^39^ Future studies should examine multiple dimensions of information search activities and vaccination intention by using multidimensional psychometric measures.^40,41^ Third, our participants were mostly white and had college degrees or more. Hence, our findings should be interpreted with caution and may not generalize across all races and education levels. Finally, we asked about information exposure in the past 3 days without capturing search activities outside that. Although 3 days have been suggested as a time period for respondents to provide accurate information with minimal recall bias,^42^ future studies should capture a longer time span of information search.

## Conclusions

In conclusion, our study examined the association between the content of COVID-19 information search activities and their vaccination intention. People who searched for information about the science of viral effects of COVID-19 and policies/procedural recommendations also reported higher vaccination intention. Risk communication should meet the consumer’s information needs by prioritizing the dissemination of COVID-19 vaccination information, specifically the science behind vaccines, and clarifying how recommended policies/procedures can reduce risk.
